# Neoplastic cells are a rare component in human glioblastoma microvasculature

**DOI:** 10.18632/oncotarget.427

**Published:** 2012-01-30

**Authors:** Fausto J. Rodriguez, Brent A. Orr, Keith L. Ligon, Charles G. Eberhart

**Affiliations:** ^1^ Department of Pathology, Johns Hopkins University; ^2^ Department of Pathology, Brigham and Women's Hospital and Harvard Medical School; ^3^ Department of Medical Oncology and Center for Molecular Oncologic Pathology, Dana-Farber Cancer Institute

**Keywords:** Glioblastoma, microvascular proliferation, endothelium, stem cells, FISH

## Abstract

Microvascular proliferation is a key biological and diagnostic hallmark of human glioblastoma, one of the most aggressive forms of human cancer. It has recently been suggested that stem-like glioblastoma cells have the capacity to differentiate into functional endothelial cells, and that a significant proportion of the vascular lining in tumors has a neoplastic origin. In principle, this finding could significantly impact the efficacy and development of antiangiogenic therapies targeting the vasculature. While the potential of stem-like cancer cells to form endothelium in culture seems clear, in our clinical experience using a variety of molecular markers, neoplastic cells do not contribute significantly to the endothelial-lined vasculature of primary human glioblastoma. We sought to confirm this impression by analyzing vessels in glioblastoma previously examined using chromogenic in situ hybridization (CISH) for *EGFR* and immunohistochemistry for mutant IDH1. Vessels containing cells expressing these definitive neoplastic markers were identified in a small fraction of tumors, but only 10% of vessel profiles examined contained such cells and when identified these cells comprised less than 10% of the vascular cellularity in the cross section. Interestingly, these rare intravascular cells showing *EGFR* amplification by CISH or mutant IDH1 protein by immunohistochemistry were located in the middle or outer portions of vessel walls, but not amongst the morphologic boundaries of the endothelial lining. To more directly address the capacity of glioblastoma cells to contribute to the vascular endothelium, we performed double labeling (Immunofluorescence/FISH) for the endothelial marker CD34 and *EGFR* gene locus. Although rare CD34 positive neoplastic cells unassociated with vessels were identified (<1%), this analysis did not identify *EGFR* amplified cells within vascular linings, and further supports our observations that incorporation of glioblastoma cells into the tumor vessels is at best extremely rare, and therefore of questionable clinical or therapeutic significance.

## INTRODUCTION

Glioblastoma represents the most common primary malignant brain tumor in humans [1], with an incidence of 3.19 per 100,000 person years in the United States [2]. These are highly aggressive neoplasms, with very poor overall survival despite combined chemotherapeutic and radiotherapy regimens [3]. Recent large scale genomic studies have clarified the common somatic genetic alterations that occur in human glioblastoma. Many target well known cellular signaling pathways, including receptor tyrosine kinase/RAS/PI3K, p53 and RB [1, 4]. Specific molecular alterations affecting these pathways include amplifications and mutations in *EGFR* and *PDGFR*, as well as mutations or deletions in tumor suppressor genes including *PTEN*, *CDKN2A*, *TP53*, *RB1*, and *NF1* [5-9]. Many of these alterations affect key biological properties of glioblastoma, including proliferation and cell invasion [10]. More recently, point mutations affecting metabolic proteins such as *IDH1* or *IDH2* have been found in the majority of infiltrating gliomas and a subset of glioblastomas [11-13]. The most frequent IDH1 mutant protein (R132H) can be identified by a specific antibody using immunohistochemistry [14, 15], facilitating precise localization of tumor cells.

It has also become clear that glioblastomas are quite heterogeneous, with stem-like cells, better differentiated components, and stromal cells all playing key roles in the growth of a neoplasm [16]. Until recently, it was thought that blood vessels and other stromal elements were recruited into the growing tumor from non-neoplastic sources. Several provocative recent studies, however, have suggested that stem-like glioblastoma cells (cancer stem cells) are able to differentiate into functional vascular endothelium, and contribute significantly to the blood vessels supporting tumor growth [17-19]. If true, this would have major implications in terms of how tumor vessels are targeted therapeutically. However, based on our routine clinical practice as surgical neuropathologists vessels rarely seemed to contain mutant tumor cells and therefore we sought to perform a more formal and quantitative analysis of genetic changes in glioma vessels. We find that the contribution of neoplastic cells to tumor endothelium is small at best, and below routine detection in many tumors. Below, we review literature on the topic of angiogenesis in glioma, and present data which supports our perspective on the issue of neoplastic contribution to glioblastoma vasculature.

### Angiogenesis is a defining property of human glioblastoma

One of the most important morphologic features of glioblastoma is the presence of microvascular proliferation [20]. Indeed, such “glomeruloid” vessels are part of the histologic diagnostic criteria in the current WHO classification scheme [20]. Florid angiogenesis in glioblastoma often represents a response to hypoxia in the neoplastic microenvironment, and is frequently found surrounding areas of pseudopalisading necrosis [21]. Hypoxia leads to an increase in angiogenic factors, including VEGF [22] resulting in microvascular hyperplasia and endothelial sprouting from pre-existing vessels [21, 23]. In addition, recent studies support the induction of angiogenesis by human glioma stem cells [24], mediated in part by hypoxia [25-27], and suggest that perivascular stem cell niches can play an important role in brain tumor pathobiology [28-32]. There is an evolving literature of interactions/cross talk between glioma cells and endothelium, which involves important pathways such as the Ang1/Tie2 signaling axis [33, 34].

The importance of adequate vasculature and blood flow in glioblastoma and other tumors has resulted in the development of a number of novel therapies and laboratory approaches that target angiogenesis [35-37]. This is conceptually simpler than targeting tumor cells, as the microvasculature was long thought to be non-neoplastic and to lack genetic instability often associated with treatment resistance [38]. In the field of neurooncology, bevacizumab, a humanized monoclonal antibody against VEGF-A, represents an approved treatment strategy for recurrent glioblastoma [39-41].

### Vasculogenic mimicry

In addition to conventional angiogenic mechanisms, alternative ways to increase vascular delivery to human tumors have been proposed, including incorporation of endothelial precursors from bone marrow [42-44] and “vasculogenic mimicry” by tumor cells. The phenomenon of vasculogenic mimicry, whereby neoplastic cells create channels lacking endothelium that conduct fluid [45], has been explored in a variety of cancer types, including melanoma, carcinomas, sarcomas, and even glioblastoma [46-51]. Several investigators have described the presence of neoplastic, non-endothelial lining in such structures by a variety of morphologic and molecular methods [52-54].

In glioblastoma the phenomenon of vasculogenic mimicry was recently studied by El Hallani et al., who described the formation of vascular-like channels in tissue sections from human glioblastoma [47]. They differed from true vessels by lack of CD34 expression in luminal cells demonstrating *EGFR* amplification by Fluorescence In Situ Hybridization (FISH), supporting the concept that these cells are not endothelial. Follow-up *in vitro* studies using CD133+ stem-like cells cultures from glioblastomas demonstrating channel formation, resulted in tubular structures in 3D matrigel, and expression of genes associated with vasculogenic mimicry (e.g. EphA2, laminin, neuropilin-2), but a consistent lack of endothelial marker expression. Interesting as these results may be, the extent of the potential contribution of vasculogenic mimicry to blood flow to glioblastoma in unclear at the present time.

### Neoplastic contribution to endothelial-lined microvasculature in glioblastoma?

Studies from several investigators have raised the possibility that true endothelial linings may have a neoplastic origin in some human cancers, including lymphoma and neuroblastoma [55, 56], and contain cytogenetic aberrations in cultures from primary tumors and in xenograft model systems [57, 58]. The capacity of tumor-derived stem cells to differentiate into endothelium and participate in the formation of microvasculature has also been studied recently in breast cancer [59]. Finally, it has been suggested that non-neoplastic neural stem cells can differentiate into endothelial cells *in vitro* [60].

In recent elegant studies using *in vitro* systems, transgenic models and primary human tumors [17-19], the hypothesis that neoplastic cells in glioblastoma contribute to the microvasculature by differentiating into endothelial cells was further explored. These groups described the differentiation of stem-like glioblastoma cells into endothelium *in vitro*, and their incorporation into tumor-associated blood vessels *in vivo*. This represents an interesting extension of the cancer stem cell hypothesis, and opens the possibility that a small fraction of multipotent cells may give rise not only to the main tumor mass [61, 62], but also its microvasculature.

In one of these studies, Ricci-Vitiani et al. found CD31 positive endothelial cells to label with p53 protein, and in double immunofluorescence/FISH experiments reported alterations in chromosomes 10, 19q, and 22q in neoplastic cells and a subset of endothelial cells. Follow-up quantification experiments using freshly dissociated glioblastomas demonstrated tumor-specific cytogenetic aberrations in between 20-90% of sorted putative endothelial cells. Cultured glioblastoma stem-like cells differentiated into endothelial cells *in vitro* and produced tumors in xenografts containing human-derived endothelial cells. Using similar approaches, Wang et al. demonstrated *EGFR*/Chromosome 7 abnormalities in CD105+ putative endothelial cells obtained from human glioblastomas, as well as endothelial differentiation from CD133+ tumor stem-like cells, thought to occur through a CD133+/CD144+ endothelial progenitor. Importantly, this second group also felt that neoplastic cells contributed significantly to the glioblastoma vasculature in human specimens, and stated that the proportion of CD105+ endothelial cells with extra copies of EGFR or chromosome 7 was comparable to the proportion of tumor cells with the same aberrations.

A subsequent study by Soda et al. predominantly used a murine glioblastoma model expressing GFP, H-ras, and Akt, coupled with p53 loss, in GFAP+ cells [19]. In these animals a subset of tumor vasculature co-expressed GFP and endothelial markers. Additional functional experiments suggested that the endothelial differentiation was induced by hypoxia, and resistant to anti-VEGF therapy. Collectively, these studies suggest a new paradigm in the field of angiogenesis: namely that genetically altered tumor cells help form their own microvasculature. These concepts have important scientific therapeutic implications, highlighted by the preliminary investigations of the effects of anti-VEGF therapy on neoplastic endothelium in two of the above studies [17, 19].

### Lack of endothelial neoplastic molecular changes in clinical glioblastoma specimens

While even a limited cancer stem cell contribution to the vasculature of glioblastoma would be of basic biological interest, for such a process to be of functional significance in therapeutic planning the tumor-derived cells would need to comprise a sizable fraction of the vascular endothelium. As clinical neuropathologists, we routinely examine glioblastoma and other brain tumors with prominent neovascularization using a variety of markers. In our experience, tissue based techniques such as FISH, Chromogenic In Situ Hybridization (CISH) and immunohistochemistry consistently show molecular abnormalities to be limited to non-vascular tumor cells. This clinical experience suggests that while stem-like glioblastoma cells may have the capacity to differentiate into endothelium, they are unlikely to play a major role in forming the pathological vasculature in patients with brain tumors as suggested in recent reports.

**Figure 1:**
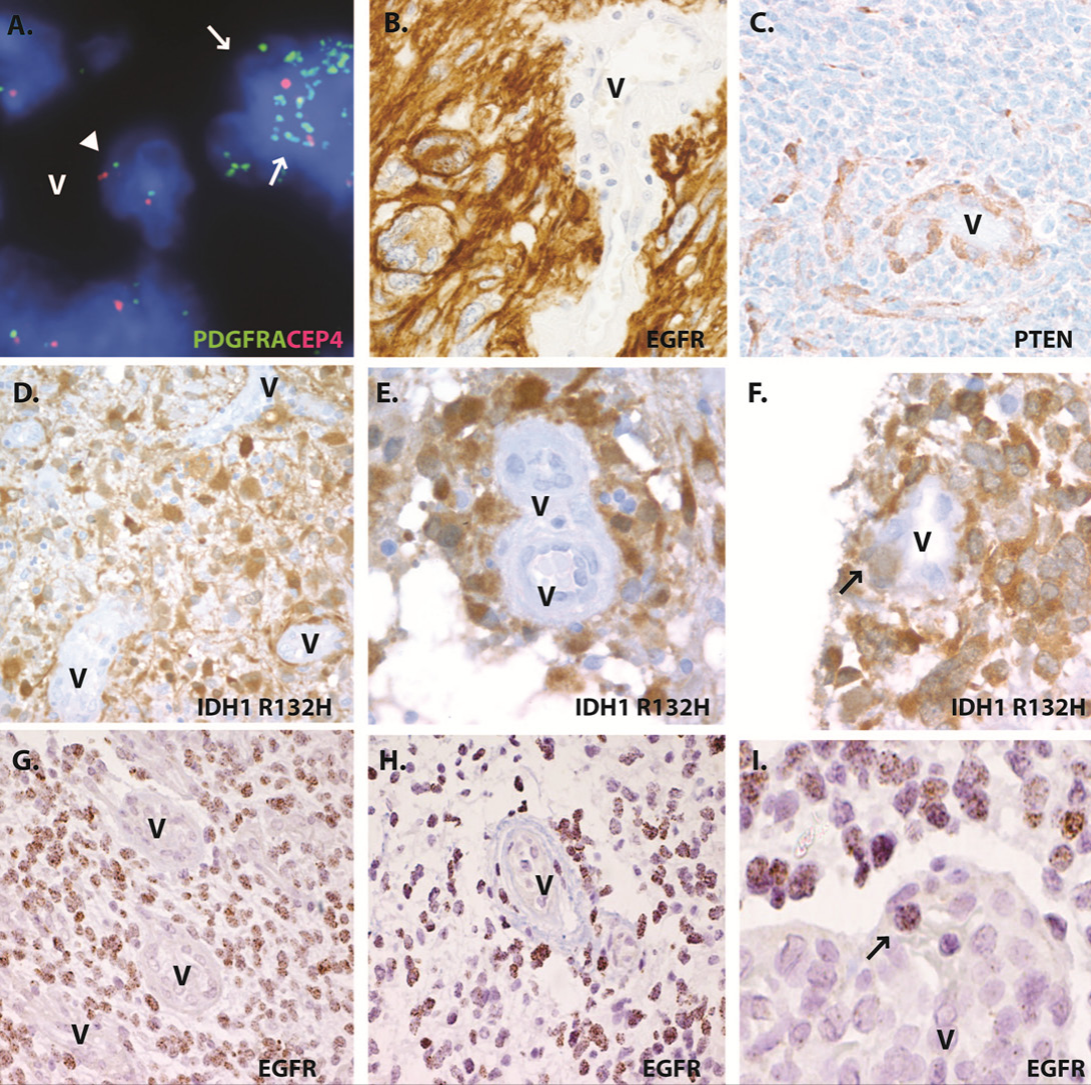
Glioma specific molecular alterations are not a common feature of endothelial tumor vasculature in clinical samples (a) ***PDGFRA*** amplification in tumor cells (arrow) but not in endothelial cells (arrowheads) in adjacent vessel (V). (b and c) Molecular alterations in neoplastic cells by immunohistochemistry, but not in vasculature (V), including EGFR overexpression (b) and PTEN loss (c). Quantification of vessels in tumors stained for IDH1 R132H mutant protein by immunohistochemistry (d-f) or ***EGFR*** amplification by CISH (g-i) revealed that most tumor cells showed positive staining, whereas the vessels (V) were devoid of signal (d and g). Tumor cells were found to crowd the perivascular region (e and h), but only rare vessels contained positive cells within the vascular wall (f and i).

Markers we have examined in glioblastomas include *EGFR*, *PDGFRA*, and *PTEN* copy number alterations using FISH or CISH, as well as alterations in IDH1, EGFR, p53 or PTEN protein using immunohistochemistry. Significantly, in our routine clinical work we have not encountered tumors in which neoplastic molecular changes were common in cells within the morphologically-defined vascular components of glioblastoma. Indeed, the tumor vasculature is routinely used as an internal negative control in such studies. Examples of normal staining in glioblastoma vessels are shown in Figure 1, including intact *PDGFRA* copy number in vessels with surrounding tumor demonstrating amplification of the receptor (Figure 1a), and overexpression of EGFR or loss of PTEN protein in glioblastoma cells with vascular structures failing to show such changes (Figure 1b,c). Such experience, while “anecdotal”, includes the examination of thousands of tumors by the authors, all of whom are board-certified neuropathologists.

To augment our clinical impressions, we have recently reviewed a number of cases using several techniques. First, we examined 24 malignant gliomas which showed strong immunoreactivity for mutant IDH1, including 9 GBM, 13 anaplastic astrocytomas, and 2 anaplastic oligodendrogliomas identified on two glioma tissue microarrays generated at Johns Hopkins Hospital. The three to four cores per tumor on the microarrays included between 4 and 51 vessel profiles (mean 18 vessel profiles per tumor), all of which were closely examined for mutant IDH1 protein similar to that seen in the surrounding infiltrating tumor cells. Most vessels showed no immunoreactivity for mutant IDH1 (Figure 1d). As illustrated in Figure 1e, glial-appearing IDH1-immunoreactive cells were found surrounding vascular structures in many tumors, consistent with the known predilection of infiltrating gliomas to satellite around blood vessels [20]. However, cells expressing this neoplastic marker were only found within the vessel wall in 1 of the 24 cases (4%). The case contained two vessels with single IDH1-positive cells within the vascular wall (Figure 1f). Importantly, in none of the tumors did we identify IDH1 positive cells definitively lining the vascular lumen, and all such endothelial cells were non-neoplastic based on this marker.

We also re-reviewed 10 glioblastoma cases from Brigham and Women's Hospital with high level *EGFR* amplification based on clinical CISH analysis. Ten random vascular profiles were scored in each tumor, most of which represented microvascular proliferations, although some normal-appearing vessels were analyzed as well. In 6 of the tumors examined, no *EGFR*-amplified cells were present within or attached to blood vessels (Figure 1g). In 3 glioblastomas, rare *EGFR*-amplified cells (1 cell in 1/10 vessels) were identified attached to the outer aspect of tumor vasculature but not definitively within the wall (Figure 1h). Finally, in one tumor, a few neoplastic cells were identified within the vessel wall (Figure 1i). In these latter 4 cases with *EGFR*-amplified cells in or around blood vessels, the percentage of vascular or perivascular neoplastic cells was always less than 10% of the overall blood vessel cellularity. Also, as with IDH1 staining, no cells lining the vessels showed increased *EGFR* copy number using CISH, supporting the concept that the endothelial lining was not derived from the surrounding tumor.

To more directly address the contribution of neoplastic cells to vascular endothelium, we concurrently examined *EGFR* gain and endothelial marker expression in a cohort of 86 glioblastomas on the Johns Hopkins tissue microarrays. These combined immunofluorescence/FISH studies were performed using an anti-CD34 antibody with a FITC labeled secondary antibody, and FISH probes targeting *EGFR*/centromere 7. Only CD34+ endothelial cells (i.e. present in the lining of a vascular lumen) were studied. All fields were scanned for vessels that demonstrated clear CD34 immunoreactivity and *EGFR* co-hybridization. Cytogenetic abnormalities in tumors were scored using previously published cutoffs [63].

**Figure 2:**
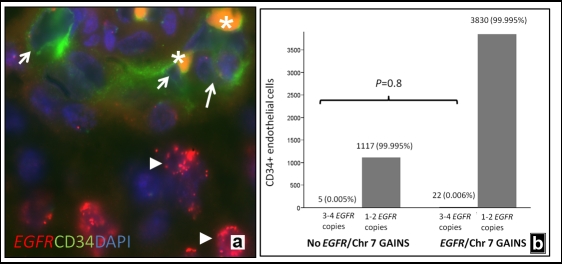
Lack of significant *EGFR* alterations in CD34 positive endothelial cells *EGFR* amplifications in glioblastoma neoplastic cells (arrowheads), but lacking in associated CD34+ endothelial cells (arrows)(a). Orange autofluorescence frequently identified intraluminal red blood cells (asterisks). Only a minority of CD34+ cells demonstrated extra *EGFR* copies, and at a similar frequency in glioblastomas with and without chromosome 7/*EGFR* abnormalities (b).

Among the 86 tumors, 32 (37%) showed *EGFR* amplification, and an additional 35 (41%) gain of chromosome 7. A total of 4974 CD34+ cells in 971 vessels were examined in the 86 glioblastomas, including 3852 CD34+ cells in 768 vessels from tumors showing *EGFR* amplification or gain of chromosome 7. No *EGFR* amplified/CD34+ endothelial cells were identified in any case. The majority of the CD34+ endothelial cells contained 1-2 *EGFR* copies (Figure 2a), with only 27 cells (0.005%) containing 3-4 copies. The number of CD34+ endothelial cells with extra *EGFR* copies was similar in glioblastomas with chromosome 7 gain or *EGFR* amplification and the smaller subset lacking such abnormalities (p=0.8, Fisher Exact Test). This suggests that random nuclear overlap may explain the low-level extra *EGFR* copy numbers we observe in rare cells.

## DISCUSSION

The plasticity of glioblastoma has long been recognized, and until recently this tumor was known as “glioblastoma multiforme” in recognition of its varied cellular composition. Divergent differentiation in the form of mesenchymal elements, including neoplastic bone [64], cartilage [65], skeletal muscle [66], smooth muscle [67], and adipose tissue [68], as well as epithelial structures [63] have all been described in glioblastoma, particularly the subtype known as “gliosarcoma”. It is also increasingly clear that glioblastoma and other malignancies contain stem-like cells which can be maintained in serum-free media as spheres and have the potential to differentiate along a number of lineages. We therefore do not doubt one central finding of recent elegant studies by several groups - that malignant glioma cells have the capacity to express endothelial markers and incorporate into blood vessels [17-19].

However, as practicing neuropathologists who routinely assess a variety of molecular markers in primary human tumors, we do question the degree to which this phenomenon actually occurs in patients. Our data suggest that very few vascular cells in human glioblastoma harbor the molecular changes which define the surrounding tumor. These results are consistent with a prior microdissection-based study, in which glomeruloid p53 immunoreactive endothelial cells in glioblastoma lacked tumor-specific mutations [69].

A number of potential caveats apply to the study of neoplastic endothelium. First, it is possible that small groups of tumor cells which do not form morphologically-recognizable vessels may express endothelial markers, and that such cells contributed to those isolated by Wang et al. [17] and Ricci-Vitiani et al. [18] using flow cytometric approaches lacking spatial cues. In addition, because of the intimate relationship between glial tumor processes and adjacent blood vessels, it can sometimes be difficult to unambiguously distinguish glial tumor cells from vasculature particularly as obtaining single cell suspensions is challenging in brain tumors. Another consideration would be that GBM cells are able to transmigrate across the vessel wall in order to enter into the bloodstream and might therefore be observed during their migration. Finally, genetic heterogeneity exists in glioblastoma, including mosaic amplification of receptors such as *EGFR* [70], potentially complicating distinctions between neoplastic and non-neoplastic cells.

It is possible that small numbers of neoplastic cells contribute to the endothelium of glioblastoma. Indeed, as shown in Figure 1, rare cells within the vessel walls can express molecular markers of neoplastic transformation. However, in neither our large anecdotal clinical experience nor the more focused analysis described above have we observed a significant contribution by neoplastic cells to the tumor microvasculature as suggested in recent reports. Specifically, we feel that most glioblastoma vessels contain no neoplastic cells, and when they are present they comprise less that 10% of the vessel cellularity. It also appears to us that the rare neoplastic cells within tumor vessels do not incorporate into the endothelial vascular lining or express the endothelial marker CD34. We therefore believe that while recent demonstrations of cancer stem cell plasticity are of significant basic research interest, the clinical and *in vivo* biological relevance of the capacity of stem-like glioblastoma cells to give rise to endothelium remains unclear, and current efforts to exploit the genotypic/phenotypic differences between tumors and their associated vasculature should not be abandoned.
